# Alcohol branding during rugby union matches in Ireland after commencement of Sect. 15 from the Public Health (Alcohol) Act: a frequency analysis of highlights from the European Rugby Champions Cup and Six Nations Championship

**DOI:** 10.1007/s11845-023-03331-8

**Published:** 2023-03-21

**Authors:** Nathan Critchlow, Richard I. Purves

**Affiliations:** Institute for Social Marketing and Health, Faculty of Health Sciences and Sport, University of Stirling, Stirling, FK9 4LA Scotland

**Keywords:** Alcohol marketing, Alcohol sponsorship, Alcohol advertising, Alcohol promotion, Public Health (Alcohol) Act, Sponsorship

## Abstract

**Background:**

Under Sect. 15 of the Public Health (Alcohol) Act 2018, Ireland has banned alcohol advertising in or on the sports area during a sports event, except for branded clothing. The restrictions commenced on 12th November 2021, but concerns have been raised that alcohol branding continues to feature in the now-prohibited sporting area.

**Aim:**

To examine the frequency and nature of alcohol brand references in or on the sporting area during two rugby union tournaments played in Ireland after Sect. 15 had commenced.

**Methods:**

A frequency analysis recorded visual references to alcohol brands in or on the sporting area (lasting ≥ 1 s) during highlights of fixtures played in Ireland during the 2021/2022 European Rugby Champions Cup (*n* = 11 matches; ‘ERCC’) and 2022 Six Nations Championship (*n* = 3 matches). Highlights were obtained from the official YouTube channels of each tournament.

**Results:**

Across both tournaments, 481 alcohol brand references were observed in or on the sporting area (ERCC = 420; Six Nations = 61). Most references were advertising for zero-alcohol variants (ERCC = 77.1%; Six Nations = 83.6%) but using similar brand iconography as their ‘regular-strength’ counterparts (e.g. brand names and logos). The remaining references were classified as alibi marketing for ‘regular-strength’ alcohol products (ERCC = 22.9%; Six Nations = 16.4%), as alcohol brand logos were presented without explicit reference to a zero-alcohol variant.

**Conclusions:**

Alcohol branding continued to feature in or on the sporting area after the commencement of Sect. 15 of the Public Health (Alcohol) Act. Clarification is needed over whether the promotion of zero-alcohol products and alibi marketing is compatible with Sect. 15 of the Act.

## Introduction

Televised sporting events provide a high-reach and salient opportunity to market alcohol [1, 2], and exposure to alcohol sports sponsorship is positively associated with consumption [3]. Accordingly, alcohol branding features frequently during live sports played in Ireland [4, 5], and this is reflected in consumer recall, including among young people [6–8]. Since the 12th November 2021, Sect. 15 of Ireland’s Public Health (Alcohol) Act 2018 (hereafter ‘the Act’) has made it an offence to advertise an alcohol product in or on the sports area during a sports event (e.g. logos printed or imposed on the pitch), with the exception of branded clothing (e.g. logos on shirts worn by players or officials) [9, 10]. Despite these restrictions, Alcohol Action Ireland, an advocacy organisation, has raised concerns that alcohol branding continues to appear within the now-prohibited sporting area [11, 12]. This study therefore examines the frequency and nature of alcohol brand references in or on the sporting area during two rugby union tournaments played in Ireland after the Sect. 15 restrictions had commenced.

## Methods

### Design

A frequency analysis was conducted on visual references to alcohol brands that appeared in or on the sporting area during highlights of fixtures played in Ireland during two rugby union tournaments: the 2021/2022 European Rugby Champions Cup and the 2022 Six Nations Championship. The design was informed by previous studies of marketing during televised sports [1, 13–15], including previous iterations of the Six Nations Championship [5].

### Tournaments and fixtures sampled

Video highlights were identified for fixtures played in Ireland during the 2021/2022 European Rugby Champions Cup (*n* = 11 matches, hereafter ‘ERCC’) and the 2022 Six Nations Championship (*n* = 3 matches) (Table 1). The ERCC is a continental tournament contested by club teams, with Ireland represented in 2021/2022 by Leinster Rugby, Munster Rugby, and Connacht Rugby. Although Ulster Rugby, another provincial team from the island of Ireland, also featured in the 2021/2022 ERCC, their home fixtures are played in Belfast, Northern Ireland, where the Sect. 15 restrictions do not apply. The Six Nations Championship is an international tournament which includes Ireland among the competitors. Both competitions were sponsored by alcohol companies. Heineken were the lead sponsors of the ERCC—with the tournament branded as the ‘Heineken^®^ Champions Cup’ [16]—and Guinness were the title sponsor of the Six Nations Championship [17].

**Table 1:** Fixtures sampled from the Six Nations Championship and European Rugby Champions Cup (ERCC)

**Tournament/match**	**Date**	**Venue**	**Length (mm:ss)**	**Views**^**a**^
***Six Nations Championship***				
Ireland vs. Wales	5th February 2022	Aviva Stadium, Dublin	05:01	266,237
Ireland vs. Italy	27th February 2022	Aviva Stadium, Dublin	05:34	172,958
Ireland vs. Scotland	19th March 2022	Aviva Stadium, Dublin	05:37	194,380
***European Rugby Champions Cup***^**b, c**^				
Leinster Rugby vs. Bath Rugby	11th December 2021	Aviva Stadium, Dublin	07:09	104,727
Connacht Rugby vs. Stade Français Paris	12th December 2021	The Sportsground, Galway	07:09	73,661
Munster Rugby vs. Castres Olympique	18th December 2021	Thomond Park, Limerick	05:54	84,748
Connacht Rugby vs. Leicester Tigers	15th January 2022	The Sportsground, Galway	07:00	100,995
Leinster Rugby vs. Montpellier Hérault Rugby	16th January 2022	RDS Arena, Dublin	07:12	95,372
Munster Rugby vs. Wasps	23rd January 2022	Thomond Park, Limerick	06:35	89,931
Connacht Rugby vs. Leinster Rugby	8th April 2022	The Sportsground, Galway	07:51	65,321
Leinster Rugby vs. Connacht Rugby	15th April 2022	Aviva Stadium, Dublin	09:38	103,059
Munster Rugby vs. Exeter Chiefs	16th April 2022	Thomond Park, Limerick	07:42	164,036
Munster Rugby vs. Stade Toulousain	7th May 2022	Aviva Stadium, Dublin	12:52	245,066
Leinster Rugby vs. Stade Toulousain	14th May 2022	Aviva Stadium, Dublin	07:15	244,375

*mm:ss* minutes:seconds

^a^As reported on YouTube, correct as per date of coding

^b^Leinster Rugby also competed in the final of the ERCC (vs. Stade Rochelais) but this was played a neutral venue (Stade Vélodrome, Marseille, France) where Ireland’s Sect. 15 restrictions do not apply

^c^Matches played by Ulster Rugby are not included as their home matches are played in Belfast, Northern Ireland, where the Sect. 15 restrictions do not apply

Highlights of the fixtures played in Ireland during both tournaments were obtained from the official YouTube channel of each tournament [18, 19]. Highlights were chosen because their short format made it possible to sample multiple tournaments, teams, stadiums, and cities in a time- and resource-efficient manner, thus increasing representativeness. Highlights also focus on the key match events, thus meaning the footage likely coincides with peak audience attention during the original broadcasts. If there was an option for regular or ‘extended’ highlights, the former was chosen for consistency.

### Defining alcohol brand references

Building on previous research [1, 5, 15], a reference was defined as a visual reference to an alcohol brand that appeared in or on the sporting area (i.e. the pitch) and lasted for approximately 1 s or more. As per Ireland’s Act, references on the clothing of match participants (e.g. players or officials) were not included. References were also not coded if they were very small in a wide-angle shot, significantly blurred through camera movement, mostly obstructed in the shot (e.g. view blocked by players), or mostly out of shot. References were counted each time they appeared, regardless of how long they lasted or whether they had been seen previously. A new reference started every time the camera angle or frame changed, even if the source remained the same (e.g. a brand reference seen first in a wide-angle shot and then in a close-up angle). If a reference disappeared for at least one second, it was counted as a new reference if it returned. If different reference formats were visible simultaneously (e.g. static logos on goalposts and on the pitch), then each was recorded as a separate reference.

### Coding of alcohol brand references

For each observed alcohol brand reference, it was recorded: (i) which tournament it related to; (ii) which fixture it related to; (iii) the time stamp it began, using the YouTube media function; (iv) the maximum number of identical references visible at the same time (e.g. multiple logos on the protective covers of the goalposts); (v) duration, in seconds; and (vi) which alcohol brand/variant was referenced. A free-text item recorded information about reference format and location. All coding was conducted by NC. Once the full sample had been coded, all coding was reviewed against the original footage a second time to ensure consistency within and between highlights.

### Analysis

All data was coded into, and analysed using, SPSS version 28. For each tournament, frequencies examined the number of alcohol brand references observed in or on the sporting area and which brands and variants featured. Medians (*Mdn*) and inter-quartile ranges (IQR) examined reference duration and the maximum number of identical references visible at the same time. Relative frequency was computed for each highlights video by dividing the video length (in seconds) by the number of references observed. Although relative frequency may not represent the exact distribution in each highlights package, it provided a standardised means of comparing videos of varied length, within and between tournaments.

## Results

### Sample characteristics

The 14 matches were played between 11th December 2021 and 14th May 2022. The length of highlights ranged from 05:01 to 12:52 (mm:ss) (Table 1). The matches covered four stadiums in three cities in Ireland (Dublin, Galway, Limerick). The cumulative number of video views (as per the date of coding) was 2,004,866 (*Mdn* = 103,893; range: 65,321 to 266,237). The cumulative number of video likes was 14,578 (*Mdn* = 745; range: 447 to 2,300 [rounded by YouTube]).

### Six Nations Championship

Across highlights of the three fixtures played in Ireland during the 2022 Six Nations, 61 alcohol brand references were observed in or on the sporting area. There were 18 in each of the highlights versus Wales and versus Scotland, and 25 in highlights versus Italy. In terms of relative frequency, this equated to a reference once, on average, every 13 s versus Italy, every 17 s versus Wales, and every 19 s versus Scotland. The median number of identical references visible at the same time was 3 (IQR: 2–4), and the median duration of references was 3 s (IQR: 2–5 s).

All alcohol brand references observed in or on the sporting area during the Six Nations were for the tournament’s title sponsor, Guinness. Most references (83.6%) related to Guinness 0.0%, as branding for this variant was featured on protective covers around the goalposts. These references featured the brand name in white font, ‘0.0’ in blue font, and the ‘gold harp’ brand logo on a black background. These references also featured branding for DrinkAware at the base, but this was only sporadically visible in close-up shots. The remaining references were coded as Guinness (16.4%). These references related to flags and flagpoles that marked the edge of the pitch, which featured the Guinness ‘gold harp’ brand logo on a black background without reference to the zero-alcohol variant. These flags and flagpoles also displayed the tournament name and logo.

### European Rugby Champions Cup

Across highlights of the 11 matches played in Ireland during the 2021/2022 ERCC, 420 alcohol brand references were observed in or on the sporting area (*Mdn* = 34; range: 20–88). Relative frequency ranged from once, on average, every 8 s in the semi-final featuring Leinster versus Stade Toulousain to once every 18 s in Munster versus Castres Olympique and Connacht versus Leinster. The median number of identical references visible at the same time was 2 (IQR: 1–4), and the median duration was 3 s (IQR: 2–5 s).

All alcohol brand references observed in or on the sporting area during the ERCC related to the tournament’s title sponsor, Heineken. Most references (77.1%) were for Heineken 0.0%, as branding for this variant featured on protective covers around the goalposts in all matches. For the quarter and semi-finals (featuring Stade Toulousain versus Muster and Leinster Rugby, respectively), there were also static adverts for Heineken 0.0% printed on the pitch behind the goal lines. References on the protective covers and pitch featured the brand name and ‘0.0’ in white font, with the ‘red star’ brand logo between them, on a blue background. The remaining references were coded as Heineken (22.9%). These references mostly related to flags and flagpoles marking the edge of the playing area. These displayed the ERCC tournament logo, which also featured the Heineken ‘red star’ brand logo in the middle, without explicit reference to the 0.0% variant. This tournament logo was also occasionally visible on the ball and, in the quarter-finals and semi-finals, on a large static logo in the centre of the pitch.

## Discussion

This analysis demonstrates that alcohol branding continued to appear in or on the sporting area during both club and international rugby union matches played in Ireland after Sect. 15 had commenced. Most references were for brand variants with zero alcohol, albeit incorporating similar brand iconography to their ‘regular strength’ counterparts. Approximately a quarter of references were coded as promoting a ‘regular strength’ alcohol product, as the brand logos were presented without explicit reference to the zero-alcohol variants. These findings build on research examining alcohol marketing in Ireland before the restrictions commenced and further contribute to understanding of how the alcohol industry responds to marketing controls [4, 5, 15, 20].

In both tournaments, advertising for zero-alcohol products used branding similar to their ‘regular-strength’ counterparts, such as the same brand names, fonts, and logos. For Guinness 0.0% branding in the Six Nations, the brand name (in white font) and ‘gold harp’ logo featured on a black background, thus creating livery akin to the branding used to promote the ‘regular strength’ variant prior to the restrictions [4, 5]. For Heineken’s 0.0% branding in the ERCC, the brand name and ‘red star’ logo featured on a blue background, rather than the green livery which is synonymous with the ‘regular strength’ product [21]. Ireland’s Act does prohibit direct and indirect advertising of an alcohol product, including through brand names and logos [9, 10]. Currently, however, it is unclear whether the display of shared brand iconography in advertising for zero-alcohol variants meets the threshold to “reasonably be regarded” as a recommendation of an [alcohol] product to the public [22]. This remains a matter for Ireland’s judicial authorities to determine, with the existing complaint from Alcohol Action Ireland about branding during the ERCC an opportunity for this to be considered [12]. Any decision will also have wider repercussions, as advertising for zero-alcohol brand variants has also been observed in other places where the promotion of ‘regular strength’ variants is now prohibited, such as on public transport [11, 22].

Legal clarification is also required for the references considered to be promoting ‘regular strength’ alcohol products. In both tournaments, such references are best described as ‘alibi marketing’ because the brand logos were presented without reference to either the brand name or the zero-alcohol brand variant. Alibi marketing has been observed in other countries with statutory alcohol marketing controls [15, 20], including during previous iterations of the Six Nations Championships [4, 5]. As per zero-alcohol advertising, clarification is needed over whether such alibi marketing is considered a direct or indirect reference to an alcohol product and, if so, whether it is “reasonably” considered to be a recommendation of said product to the public. For alibi marketing in the ERCC, the Heineken ‘red star’ brand logo was also incorporated as part of the tournament logo [23], adding a further layer of complexity when determining consumer interpretation. Moreover, as alibi references for both Guinness and Heineken mostly appeared on flags and flagpoles that marked the pitch perimeter, clarification is also required over whether this match equipment and peripheral location falls under the definition of the “sports area”.

A key limitation is that the data underestimate the volume and frequency of alcohol brand references in two ways. First, in line with the objective of examining compliance with Sect. 15, coding only focused on branding that was in or on the sporting area. The myriad other forms of marketing observed in the videos, but not coded, included: (i) advertising boards around the pitch border/stadium; (ii) branded clothing worn by players and match officials; (iii) advertising on electronic screens (e.g. scoreboards); (iv) supporters with branded merchandise or packaging; (v) graphics superimposed onto videos; and (vi) branding in the video descriptions. Second, by only focusing on highlights, the coding does not capture ‘out-of-play’ references from the original (longer) television broadcasts. In such broadcasts, the pre-match build-up and post-match analysis may have featured archive footage from previous tournaments when alcohol marketing was permitted in or on the sporting area in Ireland or footage of matches from the current tournament played in countries where such marketing is still permitted (e.g. highlights of matches from England or Scotland). The original broadcasts may have also contained advertisements for alcohol products during commercial breaks.

In conclusion, alcohol brand references continued to appear in or on the sports area during club and international rugby union fixtures played in Ireland after the commencement of Sect. 15 from the Public Health (Alcohol) Act 2018. Most references were for zero-alcohol variants that featured similar brand iconography to their ‘regular strength’ counterparts, although some references were adjudged to be alibi marketing for a ‘regular strength’ variant. Clarification is needed over the extent to which these marketing practises are compatible with Sect. 15 and how the Act defines alcohol advertising.

## Data Availability

The data and/or study materials are available upon reasonable request from the corresponding author.
